# Applications of Wireless Sensor Networks in Marine Environment Monitoring: A Survey

**DOI:** 10.3390/s140916932

**Published:** 2014-09-11

**Authors:** Guobao Xu, Weiming Shen, Xianbin Wang

**Affiliations:** 1 Lab of Ocean Remote Sensing & Information Technology, Guangdong Ocean University, Zhanjiang 524088, China; E-Mail: xuguobao@126.com; 2 Department of Electrical and Computer Engineering, The University of Western Ontario, London, Ontario N6A 5B9, Canada; E-Mail: xianbin.wang@uwo.ca

**Keywords:** wireless sensors, wireless sensor networks, marine environment monitoring, buoys, energy harvesting

## Abstract

With the rapid development of society and the economy, an increasing number of human activities have gradually destroyed the marine environment. Marine environment monitoring is a vital problem and has increasingly attracted a great deal of research and development attention. During the past decade, various marine environment monitoring systems have been developed. The traditional marine environment monitoring system using an oceanographic research vessel is expensive and time-consuming and has a low resolution both in time and space. Wireless Sensor Networks (WSNs) have recently been considered as potentially promising alternatives for monitoring marine environments since they have a number of advantages such as unmanned operation, easy deployment, real-time monitoring, and relatively low cost. This paper provides a comprehensive review of the state-of-the-art technologies in the field of marine environment monitoring using wireless sensor networks. It first describes application areas, a common architecture of WSN-based oceanographic monitoring systems, a general architecture of an oceanographic sensor node, sensing parameters and sensors, and wireless communication technologies. Then, it presents a detailed review of some related projects, systems, techniques, approaches and algorithms. It also discusses challenges and opportunities in the research, development, and deployment of wireless sensor networks for marine environment monitoring.

## 1. Introduction

A wireless sensor network (WSN) consists of a number of dedicated sensor nodes with sensing and computing capabilities, which can sense and monitor the physical parameters and transmit the collected data to a central location using wireless communication technologies. A WSN has a number of inherent characteristics including uncontrollable environments, topological constraints, and limited node resources for energy and computational power [1]. Generally, a WSN deploys more sensors than the optimal placement in order to improve the system reliability and the fault tolerance [2].

During the last decade, WSNs have been widely utilized in a variety of application fields related to water monitoring [3–5], forest monitoring [6,7], industrial monitoring [8,9], agriculture monitoring [10,11], battlefield surveillance [12,13], intelligent transportation [14,15], smart homes [16,17], animal behavior monitoring [18,19], and disaster prevention [20,21]. This technology can certainly be applied to the monitoring of marine environments.

On the other hand, with the development of society and economy, more and more people have started to pay attention to the marine environment. Marine environment systems are particularly vulnerable to the effects of human activities related to industry, tourism and urban development [22]. Traditionally, oceanographic research vessels were used to monitor marine environments, which is a very expensive and time-consuming process that has a low resolution both in time and space. For marine environment research, a WSN-based approach can dramatically improve the access to real-time data covering long periods and large geographical areas [23]. According to Tateson *et al.* [24], a WSN-based approach is at least one order of magnitude cheaper than a conventional oceanographic research vessel.

In a WSN-based marine environment monitoring system, various kinds of sensors are used to monitor and measure different physical and chemical parameters such as water temperature, pressure, wind direction, wind speed, salinity, turbidity, pH, oxygen density, and chlorophyll levels.

While the development and deployment of an adaptive, scalable and self-healing WSN system need to address a number of critical challenges such as autonomy, scalability, adaptability, self-healing and simplicity [25,26], the design and deployment of a lasting and scalable WSN for marine environment monitoring should take into account the following challenges different from those on land [22]:

(1) *Higher water resistance:* Sensor nodes of a marine monitoring system require greater levels of water resistance;
(2) *Stronger robustness:* A marine monitoring system needs stronger robustness, since the marine environment with waves, marine currents, tides, typhoons, vessels, *etc.*, is aggressive and complex, and causes movement of nodes;
(3) *Higher energy consumption:* Energy consumption is higher due to long communication distances and an environment in constant motion;
(4) *More unstable line-of-sight*: The oscillation of the radio antenna can cause a more unstable line-of-sight between transmitters and receivers [27].
(5) *Other problems:* There are also some other problems including the difficulty for deployment and maintenance of nodes, the need for buoy and mooring devices, sensor coverage problems [2], and possible acts of vandalism.

There have been a few literature reviews on Wireless Sensor Networks for marine environment monitoring. Albaladejo *et al.* [22] provided a comprehensive review of the research and development of oceanographic monitoring using wireless sensor networks and pointed out the challenges and difficulties of WSNs for oceanographic monitoring. This paper is intended as an update and extension of Albaladejo *et al*'s review [22] based on recent developments in this area during the past five years. The limitations and challenges of wireless sensor networks for environmental research were discussed in [28]. They reviewed several WSN applications such as water ecosystems, forest monitoring, precision agriculture, wildlife observation, disaster prevention and urban monitoring.

This paper provides a comprehensive review of recent developments in the related fields, discusses major technical challenges, and identifies future research directions. The rest of the paper is organized as follows: Section 2 briefly describes fundamentals of WSN-based marine environment monitoring systems. Section 3 reviews some related projects, systems, and technologies. Section 4 highlights various challenges and opportunities including oceanographic sensors protection, advanced buoy design, energy harvesting system design, and WSN-based system stability and reliability. Section 5 provides some concluding remarks.

## 2. Overview

This section provides an overview on the application of WSNs in marine environment monitoring, including different application areas, a common architecture of WSN-based marine monitoring systems, a general architecture of an oceanographic sensor node, sensing parameters and sensors, and related wireless communication technologies.

### 2.1. Application Areas

WSN-based marine environment monitoring has a broad coverage including a number of application areas: water quality monitoring, ocean sensing and monitoring, coral reef monitoring, and marine fish farm monitoring. Different application areas require different WSN system architectures, communication technologies, and sensing technologies.

A water quality monitoring system is usually developed to monitor water conditions and qualities including temperature, pH, turbidity, conductivity and dissolved oxygen (DO) for ocean bays, lakes, rivers and other water bodies. An ocean sensing and monitoring system is used to monitor ocean water conditions and other environmental parameters. A coral reef monitoring system is normally installed to monitor coral reef habitats using an autonomous, real-time and *in-situ* wireless sensor network. A marine fish farm monitoring system is developed to monitor water conditions and qualities including temperature and pH, and accurately quantify the amount of fecal waste and uneaten feed for a fish farm.

### 2.2. Common WSN Architecture

**Figure 1.**
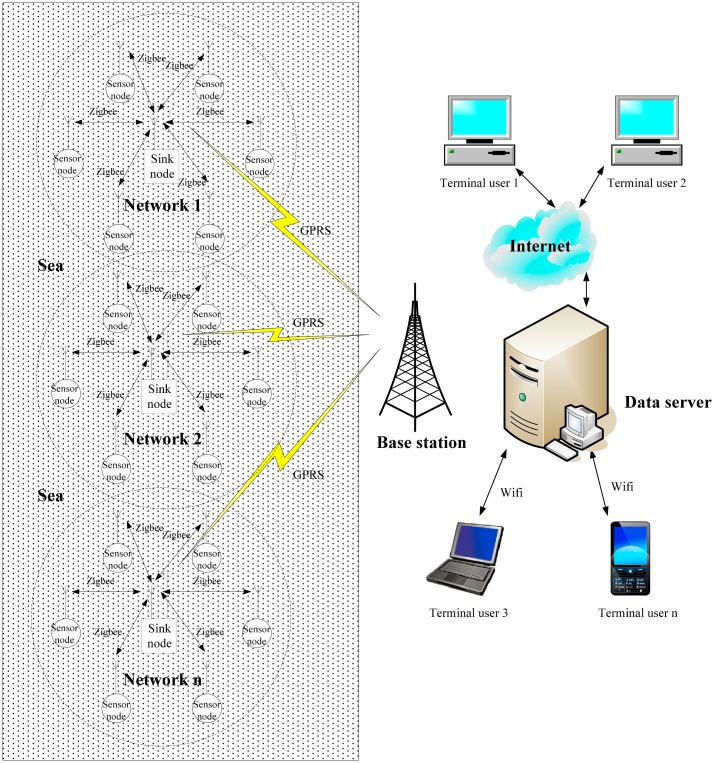
Common architecture of WSN-based marine monitoring systems.

Figure 1 shows a common wireless sensor network architecture for monitoring marine environments, which consists of sensor nodes, sink nodes, a base station, a server and user terminals. Sensor nodes can sense and monitor the *in-situ* environmental parameters such as water temperature, salinity, turbidity, pH, oxygen density and chlorophyll levels, and transmit the collected data to sink nodes via wireless communication using ZigBee or some other communication protocol. Communication between sensor nodes and a sink node is usually point-to-point. A sink node collects data from a group of sensor nodes, and transmits the collected data to the base station via the GPRS network. The server stores and processes the received data from the base station. The user terminals connect the server over the Internet.

The design and deployment of a lasting and scalable WSN for marine environment monitoring should carefully take into account the following factors: the hostile environment, the network topology, communication protocols, the number of nodes, buoys, mooring systems, oceanographic sensors, energy supply, and so on.

### 2.3. General Sensor Node

**Figure 2.**
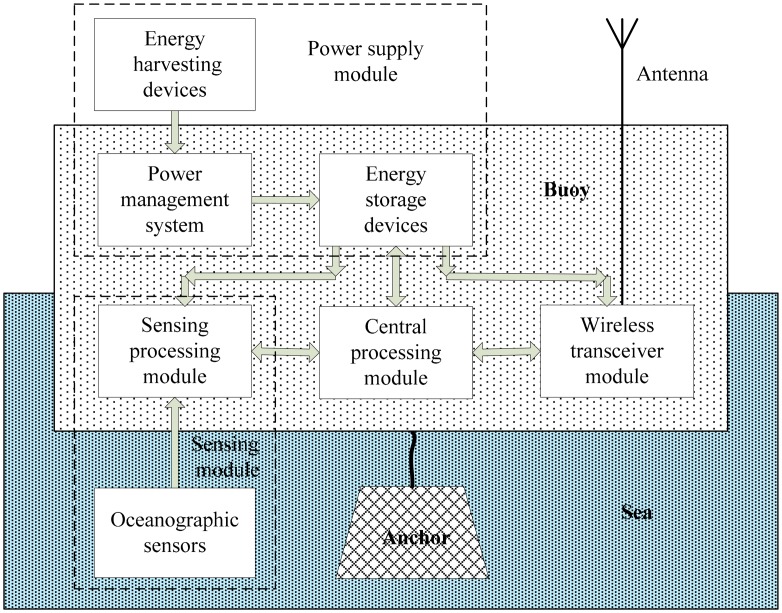
General architecture of an oceanographic sensor node.

Figure 2 shows an architecture of a general sensor node in a marine environment monitoring system. It usually includes a buoy device in order to protect electronic devices of nodes against water. A marine monitoring sensor node normally consists of the following four main modules [29]:

(1) A sensing module for data acquisition;
(2) A central processing module for local data processing and storage;
(3) A wireless transceiver module for wireless data communication;
(4) A power supply module for energy supply.

A sensing module is usually composed of several probes and sensors (with associated amplifiers and A/D converters) to sense and monitor the physicochemical parameters of marine environment as mentioned above. A central processing module normally includes a CPU and memory to process and store the collected data. A wireless transceiver module mainly consists of a RF transceiver and an antenna to send the collected data and receive instructions from the sink node. A power supply module usually contains energy storage devices (rechargeable batteries), power management system and energy harvesting devices (solar panel, wind energy, tidal power, seawater generator, *etc.*). Finally the buoy has an anchor device in order to prevent it from moving (due to waves, marine currents, wind, tide, *etc.*).

The energy options for sensor nodes usually include batteries, capacitors, heat engines, fuel cells, and energy harvesting. Sensor nodes are normally battery powered in most application systems. However, the use of a battery in sensor nodes has a number of disadvantages [30]:

(1) As sensor nodes increase in number and size, the replacement of depleted batteries is wasteful and time-consuming.
(2) A battery has limited energy that cannot last a long life for sensor nodes.
(3) Batteries have environmental contamination and disposal issues since the chemical composition of a battery often involves toxic heavy metals.

It is therefore necessary to explore an alternative power supply for sensor nodes. Harvesting energy from their ambient environment is a promising power supply for sensor networks with lower cost and long life. Energy harvesting methods include photovoltaics, fluid flow, temperature gradients, pressure variations and vibration harvesting. In terms of their efficiencies and realisability, the most outstanding energy harvesting at the moment is photovoltaics [30]. This issue will be further explored in Section 4.3.

### 2.4. Sensing Parameters and Sensors

The operating principle of sensors is to respond to changes in their environment by producing an electrical signal in the form of voltage, current, or frequency [31]. Sensors can commonly be divided into physical sensors and chemical sensors. In a marine monitoring system, physical sensors are used to measure some physical parameters, such as temperature, humidity, pressure, wind speed and wind direction, and chemical sensors are used to sense various chemical parameters (salinity, turbidity, pH, nitrate, chlorophyll, dissolved oxygen (DO), *etc.*) as shown in Table 1.

The right choice of marine environment monitoring sensors depends on the user requirements of deployment area, measurement range, accuracy, resolution, power consumption, and intended deployment time.

### 2.5. Wireless Communication Technologies

**Figure 3.**
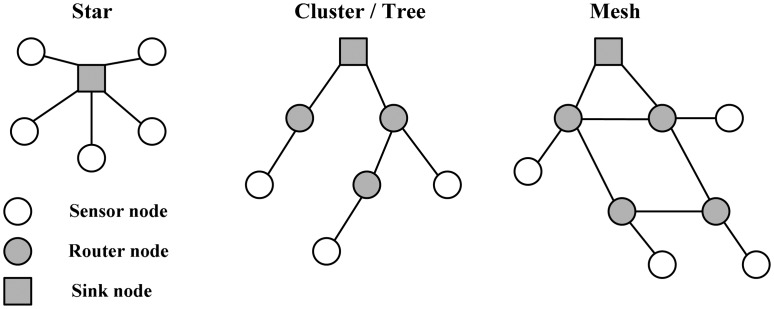
General WSN network topologies.

WSN physical topology and density are entirely dependent on the applications [32], so the design and deployment of a WSN should consider its environment and application. A number of sensor nodes are densely deployed to improve data accuracy and achieve better system connectivity. However, a dense deployment of sensor nodes has some disadvantages: high energy consumption, data collisions, interferences, *etc.* [33]. WSN nodes normally have three typical kinds of network topologies: star topology, cluster/tree topology and mesh topology, as shown in Figure 3.

(1) *Star topology:* A star topology is a point-to-point single-hop architecture in which each sensor node connects directly to a sink node. It potentially uses the least amount of power among the three topology architectures.
(2) *Mesh topology:* A mesh topology is a one-to-many multi-hopping architecture in which each router node connects to multiple nodes. Its advantages over a star topology include a longer range distance of transmission, decreased loss of data, and a higher self-healing communication ability. However, its disadvantages are at the cost of higher latency and higher power consumptions.
(3) *Cluster/tree topology:* A cluster/tree topology is a hybrid star–mesh architecture. It takes advantage of the low power consumptions and simple architecture of a star topology, as well as the extended range and fault tolerance of a mesh one. However, there probably exists some latency.

The right and reasonable choice of network topology depends on the amount and frequency of data to be transmitted, transmission distance, battery life requirements and mobility of the sensor node [34]. It should be noted that a WSN physical topology may change due to available energy, position variations of nodes, malfunction, reachability (due to noise, severe weathers, moving obstacles, *etc.*), and task details of sensor nodes [35].

A sensor node normally incorporates a radio module for wireless communication. The transmitted distance of wireless communication can be anywhere between a few meters (Bluetooth, ZigBee, WiFi, *etc.*) and thousands of kilometers (GSM or GPRS radio communication). Wireless communication has various standards and technologies including Bluetooth, ZigBee, WiFi, GSM, GPRS and WiMAX. Table 2 provides a summary and brief comparison of these communication technologies. Usually, two or more wireless communication technologies are used in a real wireless sensor network. In particular, underwater acoustic communication technologies can be a good choice for data collection and exchange among underwater sensors [36–38].

Generally, the longer the range a radio module must transmit, the more energy consumption a radio module will have. The choice of a wireless communication technology depends on the amount and frequency of the transmitted data, transmission distance, and amount of available energy.

## 3. State-of-the-Art Review

This section presents a comprehensive review of related projects, systems, applications, network routing mechanisms, algorithms, approaches and techniques on marine environment monitoring based on wireless sensor networks.

### 3.1. Related Projects, Systems and Applications

Different WSN projects, systems and applications have been proposed and developed in the literature for monitoring marine environments. Table 3 summarizes the features of related projects, systems and applications.

It can be found that most of the efforts are related to general ocean sensing and monitoring [4,23,40,44,46,47,50,53,55–57] and water quality monitoring [39,42,43,49,51,54,58]. Some specific efforts have been made for fish farm monitoring [45,48], coral reef monitoring [41], and marine shellfish monitoring [52]. Some projects focus specific technologies or devices, e.g., buoys [23,40,53,57,59] which will be further discussed in Section 4.2, and energy saving and harvesting [4,43,48,50,52,56–59], discussed in details in Section 4.3.

Most developed systems were only experimented in lab settings or indoor environments [39,45,46,52,55], some are tested in outdoor pools or small ponds/lakes [40,42,48,58], and a number of them have been tested or deployed in real marine or river environments [4,23,41,43,44,47,49–51,53,57,59].

It is also interesting to note that during the last decade most projects, systems and applications have been developed by research groups in a small number of countries, including USA [39,40,41,42], China [44,52–55], Spain [4,45,46,48,59] and Ireland [43,49].

### 3.2. Specific Networks, Routing Mechanisms and Algorithms

To satisfy the requirements for marine environment monitoring systems, researchers have proposed and developed a number of specific networks, routing mechanisms, protocols, and algorithms for WSN-based marine environment monitoring.

A WSN-based data collection framework was proposed and developed by Saha *et al.* [60] for disaster mitigation and rescue operation. A WSN communication protocol with lower delay and better energy efficiency was proposed for data dissemination from disaster areas. A simulation experiment was conducted to validate the performance of the proposed protocol comparing with the SENDROM system protocol.

A group-based underwater wireless sensor network (UWSN) was proposed by Lloret *et al.* [45] to monitor accurately the amount of fecal waste and uneaten feed deposited on the seabed which can cause the damage of the fauna and flora. The design and development of this underwater WSN took into account several factors: number of sensor nodes, sensor nodes mobility model, distribution of sensor nodes, network topology, and communication technologies.

Roadknight *et al.* [47] proposed a multi-layered scalable and adaptive approach of data management for a wireless sensor network. This algorithm consisted of three decision making components: sliding window averaging, local rules and parameter evolution. A single buoy was deployed off Scroby Sands to verify the characteristics of the proposed approach.

A WSN framework was proposed by Lu *et al.* [61] for environmental monitoring applications. Its highlight is on its network layer design by considering multiple aspects: heterogeneity, service-aware control platform, unified routing and scheduling, network monitoring. A special case study was conducted to demonstrate that the framework can be used to guide how to design a WSN for environment monitoring in the future.

Barbosa *et al.* [62] presented a routing algorithm of WSNs for marine oil slick monitoring. They proposed two methods: single relay decision (SRD) and multiple relay decision (MRD) protocols for message routing. The proposed algorithms have more efficient message distribution than single hop and greedy approaches. However, their approach does not consider node mobility, energy harvesting and network scalability.

An IEEE 802.15.4-based wireless monitoring system was presented by Loìpez *et al.* [48] to collect pH and temperature parameters in a fish farm. The proposed algorithm used a ZigBee-based routing and the application layer to manage information transmission from the source node to the central coordinator. They designed a sub-layer-based power consumption algorithm to prolong the node lifetime.

Xu *et al.* [63] proposed an improved WSN MAC protocol for marine environment monitoring to meet the demand of the energy consumption, real-time transmission, bandwidth and reliability. Simulation results show good energy consumption and network throughput abilities. However, the proposed algorithm was not implemented in the actual sensor node to verify its performance.

A WSN dedicated dynamic clustering algorithm was presented for oil slicks monitoring by Harchi *et al.* [64]. It can be applied to a monitoring system adaptively in terms of number of nodes, clustering dynamics, measurement periods, and metric weights to supervise climate conditions. Various parameters are evaluated regarding their influence on the stability of the network clustering algorithm.

Suakanto *et al.* [65] proposed a cloud computing-based approach for data processing in disaster monitoring. The proposed approach used a FTR-HTTP based delivery method from remote client to server.

Jalali *et al.* [66] proposed a cooperative hybrid ARQ (C-HARQ) mechanism in solar powered wireless sensor networks to improve energy efficiency and reliability of energy harvesting. They conducted C-HARQ experiments using a Matlab/Simulink-based simulator for networked and embedded systems. Their experimental results showed that C-HARQ is superior to C-ARQ in energy consumption of relay nodes.

### 3.3. Specific Techniques and Approaches

To address the special needs and purposes of marine environment monitoring, a number of WSN-based techniques and approaches have been developed and reported in the literature.

O'Connor *et al.* [67] presented a multi-modal event monitoring system based on WSNs and visual images for river and coastal marine detection. The system used a visual sensor to complement the use of a WSN in detecting and tracking features of a river or coastal marine location. A software tool was developed to analyze the relationship between the sensor readings and image features. It uses a support vector machine (SVM) approach for training or classification. A Matlab image processing toolbox was used for processing images and extracting various image features including color features, texture features, and edge features.

Kong *et al.* [68] designed a WSN-based water environment monitoring system which can sense and monitor video information in key areas and various water quality parameters, including water temperature, turbidity, pH, dissolved oxygen and electric conductivity. This monitoring system has a data video base station, data monitoring nodes, and a remote monitoring center. This system used an ARM-DSP based double processor, combined ZigBee and CDMA wireless transmission networks, and used a CPLD sampling controller.

A decentralized *ad-hoc* wireless sensor network was proposed for ocean pollution detection by Khan *et al.* [69]. In order to prolong the network lifetime and to improve its Quality of Service (QoS), they focused on the deployment of sensors, protocol stacks, synchronization and the routing algorithms.

A WSN-based wave monitoring technology was proposed by Marin-Perianu *et al.* [70] to monitor various wave parameters. This system deployed dense wireless sensor nodes which are equipped with low-cost, low-power, MEMS-based inertial sensors of accelerometers and gyroscopes. They conducted experiments using a Ferris wheel contraption and the results showed an accuracy of approximately 10 cm for a wheel diameter of 100 cm.

A robotic wireless sensor network was presented by Bhadauria *et al.* [71] for monitoring common carp in Minnesota lakes. This project built a small, mobile, lightweight robotic raft which is deployed with searching and tracking algorithms. They conducted several field experiments in various lakes, and experimental results demonstrated that the robotic raft has great potential in environmental monitoring. They envisioned some system improvements including energy saving, localization accuracy, autonomous navigation and multi-raft systems.

In order to enhance measurement precision and prolong the lifetime of marine environmental monitoring sensors, Delauney *et al.* [72] analyzed the biofouling effects on marine sensors measurements, proposed some promising techniques for the biofouling protection of *in situ* sensors.

To explore the impact of the deep ocean increase in CO_2_ levels and resulting pH changes on ocean biogeochemistry and ecology, Herlien *et al.* [73] studied a Free Ocean CO_2_ Enrichment (FOCE) system. The proposed algorithm can achieve the objectives of instrument-in-the-loop control, software reuse of infrastructure and instrument services, and rapid assembly of a scalable end-to-end sensor network system.

An Android-based WSN application was developed by Tembekar [74]. This WSN app can be installed and operate on any Android-based smartphone, get synchronized with the centralized database server, and monitor the various nodes of the wireless sensor network.

## 4. Research Challenges and Opportunities

So far, wireless sensor networks have been widely applied to terrestrial areas, and some of these deployments have achieved satisfactory performance. However, the application of WSNs in marine environment monitoring is still in its infancy, and most WSN-based systems are purely experimental [22]. This section discusses a few challenges of wireless sensor networks for marine environment monitoring including oceanographic sensors protection, advanced buoy design, energy harvesting system design, and system stability and reliability.

### 4.1. Oceanographic Sensors Protection

In marine environments, there are over 4000 organisms related to fouling problems [75]. According to their sizes, organisms can be classified into micro-organisms (or so called biofilms, slimes, and micro-fouling) and macro-fouling [72]. Biofouling development on a sensor surface is subject to several chemical, physical and biological factors such as pH, dissolved oxygen, temperature, light, location depth, conductivity, organic material and hydrodynamic conditions. When oceanographic sensors are immersed in seawater, they are susceptible to biofouling problems which often lead to the long-term accuracy issues of marine environmental sensor measurements. Since the marine environment is aggressive and the seawater is corrosive, oceanographic sensors should take appropriate fouling protection measures.

The biofouling protection for oceanographic sensors may be divided into three techniques according to their different actions: wipers mechanisms, copper corrosion mechanisms, and chlorine evolution mechanisms [72].

(1) *Wiper mechanisms:* A biofouling protection system based on wipers is a purely mechanical method. It is an effective biofouling protection technique as long as the sensor head has a suitable shape for wiper cleaning and the wipers are in good condition.
(2) *Copper corrosion mechanisms:* A copper corrosion mechanism is an effective biofouling protection method to protect the sensitive sensor head, but the protection mechanism is not easy to apply to existing sensors and the cost is relatively high.
(3) *Chlorine evolution mechanisms:* A biofouling protection system based on a chlorine evolution mechanism uses bleach or chlorine generation by seawater electrolysis. Moreover, this protection mechanism is easily adapted to existing sensors and the cost is relatively low.

Besides the abovementioned three biofouling protection techniques, there are some other interesting methods which promise effective results coming from research laboratories [76,77]. However, it is very difficult to implement these methods in the real sea environment.

Biofouling protection for oceanographic *in-situ* sensors is a very difficult problem. The ideal biofouling protection for oceanographic sensors should take into account six aspects: low cost, low power consumption, easy to install on existing sensors, no or low impact on measurement precision and the environment, long lifetime and robustness against aggressive conditions. Therefore, researchers and manufacturers should further study and explore the biofouling protection mechanisms for marine environmental sensors.

### 4.2. Advanced Buoy Design

Considering the marine environment is aggressive and complex, it is very crucial to design an advanced flotation device (buoy) for a marine environment monitoring system. A buoy normally consists of a wireless sensor network node (CPU, sensors, radio, and batteries), an energy harvesting module, underwater sensors and a mooring system. For example, Pirisi *et al.* [78] proposed a special energy harvesting buoy which can effectively use sea wave energy conversion as a power source and has potential applications in marine environment monitoring. Albaladejo *et al.* [59] designed a multisensory buoy system which can be effectively used for shallow marine environment monitoring.

The design and deployment of an advanced buoy for marine wireless sensor networks should take into account the following requirements: low cost, waterproof, strong stability, energy harvesting, and mooring system.

(1) *Low cost:* A marine environment monitoring system using wireless sensor networks is usually composed of a large number of sensor nodes. Therefore, each buoy device needs to be low cost.
(2) *Watertightness:* In order to protect the stability of marine environment monitoring system and prolong its lifetime, its electronic devices must be in a waterproof housing to avoid water damage.
(3) *Strong stability:* As the marine environment is aggressive and complex, the monitoring system should have a strong stability against adverse atmospheric conditions.
(4) *Energy harvesting:* Since it is not convenient to replace the batteries deployed on the marine surface and the sensor nodes, which are far away from the land and are power-hungry, it is necessary to consider the use of energy harvesting to reduce system maintenance requirements.
(5) *Mooring system:* Due to tides, waves, marine currents, wind, *etc.*, an anchor is required on the seabed in order to avoid the movement of the buoy devices.

Besides the above mentioned requirements, the buoy mechanic design should meet a number of requirements including the buoy visibility with bright yellow color and a warning light for maritime traffic, the use of environmentally friend materials, the connection of several sensors, and the reasonable antenna height for the better communication propagation.

### 4.3. Energy Harvesting System Design

The energy supply of a wireless sensor network is generally provided by batteries which have limited energy [27]. In addition, in marine environment monitoring systems, wireless sensor nodes are often deployed in unapproachable sea surface areas, and they are mostly planned for long-time operation, therefore, it is not convenient to replace the sensor batteries. Moreover, marine sensor nodes (sink nodes) have high energy consumption due to the use of long-range wireless communication protocol (GPRS). In order to reduce system maintenance requirements effectively, there is a clear need to design an energy harvesting system which uses renewable energies source such as solar [4], tidal power [78], or wind energy [79,80].

Some energy harvesting devices have been designed and developed to prolong the lifetime of marine environment monitoring systems. For example, Perez *et al.* [4] developed a solar energy harvesting device which is composed of two solar panels behind the electronic equipment with an inclination of 45 degrees in an opposite direction. In order that one battery is always being charged while the other is always being discharged, a power management system of a low-power maximum power point tracker (MPPT) circuit was developed and used for wireless sensor networks in [56] and [81].

To design an advanced energy harvesting system for marine environment monitoring, we should consider the following three aspects: energy harvesting devices, power management system, and energy storage devices.

- *Energy harvesting devices:* An energy harvesting device is responsible for harvesting energy from the ambient environment. According to the characteristics of available ambient energies, we should choose appropriate energy harvesting devices and should consider how to install the energy harvesting devices.
- *Power management system:* A power management system can intelligently manage the batteries to be charged and discharged at separate intervals of time. An ideal power management system can prolong the lifetime of batteries and easily store more energy for the system.
- *Energy storage devices:* Energy storage devices normally adopt the rechargeable batteries. Usually, the energy capacity of rechargeable batteries is larger than daily system energy consumption and daily harvesting energy in order to store energy and permit the system to supply power even in case of bad weather [56].

Given the aggressive and hostile marine environment, in order to harvest and use more reliable renewable energies, we can envision a hybrid harvesting energy system for marine environment monitoring in the future, which can use several renewable power sources such as solar, tidal power, seawater generator, and wind energy.

### 4.4. System Stability and Reliability

During the past decade, the system stability and reliability problem of wireless sensor networks has been widely studied in order to measure physical parameters correctly and effectively, as well as to prolong the lifetime of the system dramatically [82,83]. AboElFotoh *et al.* [84] studied the reliability and message delay for cooperative wireless distributed sensor networks subject to random failures. Egeland *et al.* [85] analyzed the reliability and availability of wireless multi-hop networks with stochastic link failures. Qureshi *et al.* [86] presented a methodology using a topology control mechanism for the reliability evaluation of a WSN. Silva *et al.* [8] proposed a methodology based on an automatic generation of a fault tree to evaluate the reliability and availability of wireless sensor networks in typical industrial environments.

Considering the aggressive and complex environment, it is very important to analyze the system reliability in a marine environmental monitoring system using wireless sensor networks. Therefore, the research on the reliability of a WSN-based marine environment monitoring system should take into account the following aspects.

(1) *Battery life issues:* As mentioned above, marine sensor nodes (sink nodes) consume more energy than other kinds of wireless sensor nodes. Therefore, the battery life issue always affects the system reliability.
(2) *Communication relay issue:* The communication relay affects dramatically the system reliability, when some nodes fail or simply disappear.
(3) *Severe environment conditions:* The marine environment always has external interference from ships, fishes, and birds, and has severe weather conditions such as waves, marine currents, tides and typhoons. Such severe environment conditions further influence the system reliability.

## 5. Conclusions

During the last decade, monitoring of the marine environment has attracted a great deal of research and development attention. Wireless sensor networks are a highly promising technique for monitoring marine environments because of their advantages of easy deployment, real-time monitoring, automatic operation, and low cost. This paper presents a state-of-the-art survey of applications of wireless sensor networks in marine environment monitoring. It first describes fundamentals of WSNs-based marine environment monitoring, including application areas, a common WSN architecture, a general sensor node architecture, sensing parameters and sensors, and wireless communication technologies. Then, it reviews the related literature according to different projects, systems, applications, network routing mechanisms, algorithms, approaches and techniques on marine environment monitoring based on wireless sensor networks. From this survey, it is evident that there are still a few interesting challenges and opportunities on development and deployment of wireless sensor networks for marine environment monitoring, including oceanographic sensors protection, advanced buoy design, energy harvesting system design, and system stability and reliability.

## Figures and Tables

**Table 1.** Common marine environment monitoring sensors.

**Sensors**	**Monitoring Parameters**	**Range**	**Accuracy**	**Power Supply**	**Unit**	**Manufacturer**
SBE 16plus V2	Temperature	−5 to +35 °C	±0.005 °C	9–28 V	°C	Sea-Bird Electronics
GT301	Pressure	0 to 60	< ±0.5% of FRO	24 V	bar	Kongsberg Maritime
SBE 16plus V2	Conductivity (Salinity)	0–9	± 0.0005	9–28 V	S/m	Sea-Bird Electronics
OBS-3+	Turbidity	Mud: 5000–10,000 mg/L Sand: 50,000–100,000 mg/L	0.5 NTU	15 V	NTU	Campbell Scientic
PS-2102	pH	0 to 14 pH	±0.1	N/A	pH	PASCO
YSI 6025	Chlorophyll	0 to 400 μg/L	0.1 μg/L	6 V	μg/L	YSI
ISUS V3	Nitrate	0.007 to 28 mg/L	±0.028 mg/L	6–18 V	mg/L	Satlantic
SBE 63	Dissolved oxygen (DO)	120% of surface saturation in all natural waters	0.1	6–24 V; 35 mA	mg/L	Sea-Bird Electronics

**Table 2.** Wireless communication technologies [22].

**Technology**	**Standard**	**Description**	**Throughput**	**Range**	**Frequency**
WiFi	IEEE 802.11a; 802.11b/g/n	System of wireless data transmission over computational networks.	11/54/300 Mbps	<100 m	5.8 GHz 2.4 GHz
Bluetooth	IEEE 802.15.1	Industrial specification for WPAN which enables voice and data transmission between different devices by means of a secure, globally free radio link (2.4 GHz).	v. 1.2: 1 Mbps v. 2.0: 3 Mbps UWB: 53–480 Mbps	Class 1: 100 m Class 2: 15–20 m Class 3: 1 m	2.4 GHz
ZigBee	IEEE 802.15.4	Specification of a set of high-level wireless communication protocols for use with low consumption digital radios, based on WPAN standard IEEE 802.15.4.	250 Kbps	<75 m	2.4 GHz
WiMAX	IEEE 802.16	Standard for data transmission using radio waves.	<75 Mbps	<10 km	2–11 GHz
GSM		Standard system for communication via mobile telephones incorporating digital technology	9.6 Kbps	Dependent on service provider	850/900/1800/1900 MHz
GPRS		GSM extension for unswitched (or packaged) data transmission.	56–144 Kbps	Dependent on service provider	850/900/1800/1900 MHz

**Table 3.** Summary of WSN-based marine environment monitoring projects, systems and applications.

**Reference**	**Organization**	**Country**	**Year**	**Application Areas**	**Sensing Parameters**	**Communica-tion Protocols**	**Buoy**	**Energy Harvesting**	**Testing & Deployment**	**Main Features**
Perez *et al.* [4]	Universidad Politécnica de Cartagena	Spain	2011	Ocean sensing & monitoring	Temperature, pressure, salinity, nitrates, velocity, chlorophyll, and turbidity	GPRS ZigBee	Special buoy	Two solar panels	Deployed in the harbor of Cartagena	LabVIEW-based user interface using Google Maps; Solar energy harvesting; Special buoy
Thiemo *et al.* [23]	Swedish Institute of Computer Sci. & University at Berlin	Sweden; Germany	2007	Ocean sensing & monitoring	Temperature, motion, vibration and sound	GPRS	Simple buoy and king's buoy	Batteries	Tested in Baltic Sea	Design of an advanced low-cost buoy system
Yang *et al.* [39]	Penn State University	USA	2002	Water quality monitoring	pH	RF transceiver and acoustic transducer	PVC housing	Two rechargeable batteries	Lab testing with 5 nodes	The design of various interface circuits and the use of five air-based sensor nodes
Vesecky *et al.* [40]	UC Santa Cruz	USA	2007	Ocean sensing & monitoring	Temperature, wave and location	900 MHz	mobile minibuoy	Battery power	Prototype buoy tested in a pool	An autonomous mini-buoy prototype; GPS and a PID scheme control
Bromage *et al.* [41]	UC Santa Cruz	USA	2007	Coral reefs monitoring	Temperature, pH, light, pressure, and conductivity	900 MHz	Watertight housing	Battery	Monterey Bay deployment	Programmable Oceanic Device (POD) with a 4-mode scheduler to save energy
Seders *et al.* [42]	University of Notre Dame	USA	2007	Water quality monitoring	Temperature, pH, and DO	433 MHz	Box and polyethylene ring	12 volt marine battery	Tested a prototype in a small lake	A LakeNet sensor pod and an altered sampling strategy
Regan *et al.* [43]	Dublin City University	Ireland	2009	Water quality monitoring	Temperature, pH, turbidity, DO and conductivity	ZigBee	Inshore sensor buoys	Solar panel and power pack	Deployed in five sites on the River Lee, Ireland	A real-time heterogeneous water quality monitoring; Sensor maintenance
Liu *et al.* [44]	Hong Kong University of Sci. and Tech.	China	2010	Ocean sensing & monitoring	Sea depth and temperature	ZigBee	Sensor floating	Batteries	Deployed in HKUST campus and Tsingtao	A Perpendicular Intersection (PI) mobile-assisted localization scheme
Lloret *et al.* [45]	Universidad Politecnica de Valencia,	Spain	2011	Marine fish farms monitoring	The amount of pollution	?	Buoy	?	Tested on OPNET Modeler network simulator	A group-based underwater WSN for monitoring fecal waste and uneaten feed
Macias *et al.* [46]	Universidad de Las Palmas de Gran Canaria	Spain	2011	Ocean sensing & monitoring	Visible-field, sound and temperature	ZigBee and acoustic	?	?	Tested on module of NS-3	Three tier communication architecture; transmitting video streaming data
Roadknight *et al.* [47]	University of Kent	UK	2004	Ocean sensing & monitoring	Temperature, conductivity, water depth, turbidity	?	Single buoy	Batteries	Buoy deployed off Scroby sands	A multi-layered scalable and adaptive approach of data management
Loìpez *et al.* [48]	Universitat de Barcelona	Spain	2010	Fish farm monitoring	Temperature and pH	ZigBee	?	One rechargeable battery	Tested in a pool	A sub-layer-based power consumption algorithm
O'Connor *et al.* [49]	Dublin City University	Ireland	2012	Water quality monitoring	Temperature, conductivity and depth	?	Buoys	?	Tested in River Lee, Poolbeg Marina and Galway Bay	A multi-modal environment monitoring network based on WSN and visual image
Cella *et al.* [50]	University of Queensland	Australia	2009	Ocean sensing & monitoring	Temperature and illuminance	ZigBee	Cylinder waterproof buoys	Two solar panels	Deployed in the Moreton Bay	Two solar cells and the underwater wireless communication
Diofantos *et al.* [51]	Cyprus University of Technology	Cyprus	2009	Water quality monitoring	Temperature, pressure salinity and turbidity	GPRS	Cylinder waterproof buoy	Battery	Deployed in a municipal beach	Integrating two technologies of satellite remote sensing and WSN
Yang *et al.* [52]	Zhejiang University of Technology	China	2009	Monitoring marine shellfish	Water temperature, pH value, salinity, DO and COD	GPRS	?	Solar battery	Tested in an aquatic experimental base	Multi-hop communication protocol, multiple nodes, and SMT
Jiang *et al.* [53]	Ocean University of China	China	2009	Ocean Sensing & monitoring	Temperature, velocity and light	ZigBee	Lever buoy	Battery	Deployed off the seashore	The sleep mechanism and lever buoy
Jin *et al.* [54]	China Jiliang University	China	2010	Water quality monitoring	Temperature, pH, DO, and salinity	ZigBee GPRS	?	Battery	?	Two wireless communications of ZigBee and GPRS
Chi *et al.* [55]	Shanghai Ocean University	China	2010	Ocean Sensing & monitoring	Water temperature, DO and pH	ZigBee	Buoys with GPS &PEA	?	Experimented in two testbeds	Position determination and location verification using GPS& PEA; Buoys
Cesare *et al.* [56]	Politecnico di Milano, Milano	Italy	2011	Ocean Sensing & monitoring	Seawater luminosity, temperature and moisture	ZigBee	Cylinder waterproof buoys	Solar energy harvesting	Deployed in the Moreton Bay	Optimal solar energy harvesting; Power-aware and adaptive TDMA protocol
De Marziani *et al.* [57]	National University of Patagonia San Juan Bosco	Argentina	2011	Ocean Sensing & monitoring	Temperature, pressure, PAR radiation, pH and salinity	ZigBee	Cylinder waterproof buoys	Solar panels	Tested in San Jorge Gulf	A low cost reconfigurable WSN; Buoys; Solar panels
Alkandari *et al.* [58]	Kuwait University	Kuwait	2012	Water quality monitoring	Water temperature, DO, and pH	ZigBee 802.11 Ethernet radio	?	A high-capacity solar panel	Tested in a water pool	Using ZigBee and 802.11 Ethernet radio and a high capacity solar panel
Albaladejo *et al.* [59]	Technical University of Cartagena	Spain	2012	Ocean Sensing & monitoring	Marine temperature and pressure	ZigBee	Special buoy	Solar panels	Deployed in Mar Menor Lagoon	A new multisensory buoy system and solar panels

**Notes:** ?: Related information is not available from the reference; DO: Dissolved Ox0ygen; COD: Chemical Oxygen Demand.
